# Supplementary material for: In Vitro Transcribed Artificial Primary MicroRNA for the Inhibition of Hepatitis B Virus Gene Expression in Cultured Cells
Source: Microorganisms. 2025 Mar 5;13(3):604. doi: 10.3390/microorganisms13030604 (PMC11946339; doi:10.3390/microorganisms13030604)
Supplement: Supplementary file 1 [file microorganisms-13-00604-s001.zip › microorganisms-3405186-supplementary.pdf]

IN VITRO TRANSCRIBED ARTIFICIAL PRIMARY MICRORNA FOR THE INHIBITION OF HEPATITIS B VIRUS GENE EXPRESSION IN CULTURED CELLS

SUPPLEMENTARY INFORMATION

Creanne Shrilall, Patrick Arbuthnot and Abdullah Ely

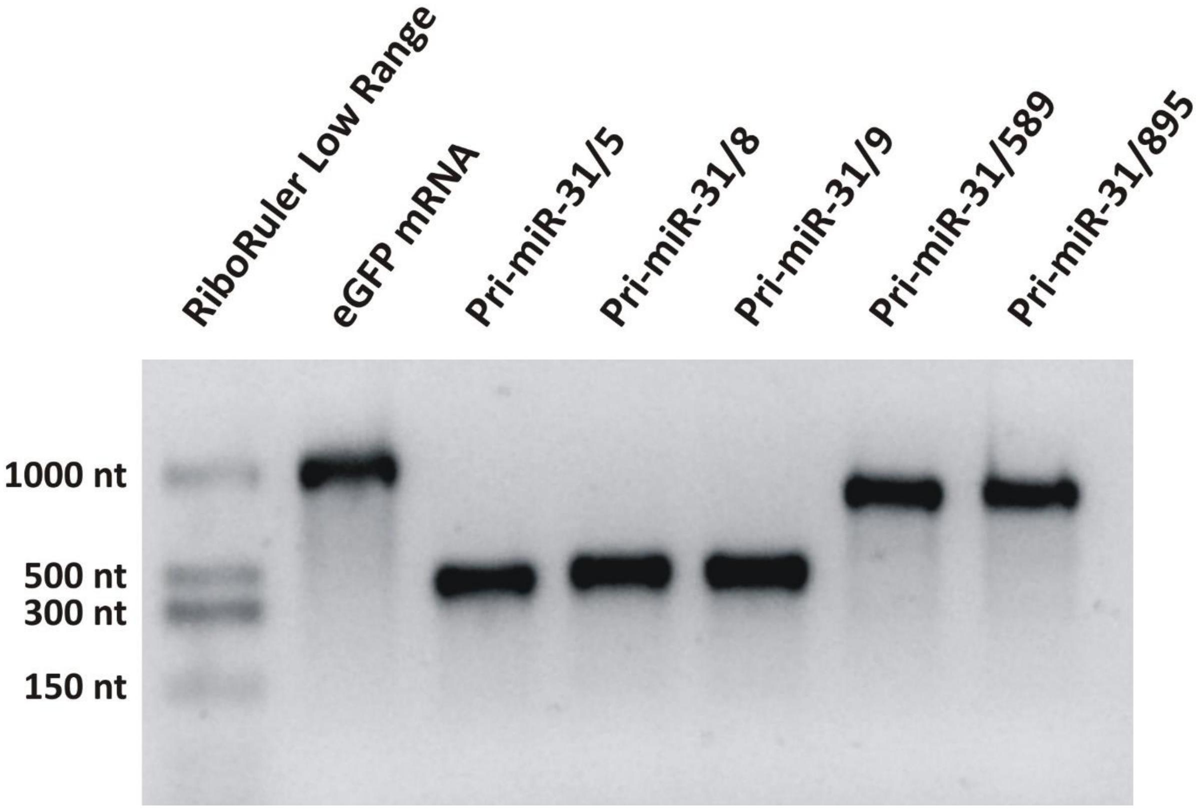

Supplementary Figure S1: In vitro transcribed RNA, capped and cellulose purified.

Linearised eGFP and pri-miR-31 vectors were used as template for in vitro transcription. The RNAs were purified by cellulose chromatography and capped enzymatically using the Vaccinia Capping Enzyme System (NEB). RNAs were resolved on a 1% formaldehyde gel (eGFP mRNA = 959 nt; pri-miR-31/5, -31/8 and 31/9 = 387 nt; pri-miR-31/589 and -31/895 = 721 nt).

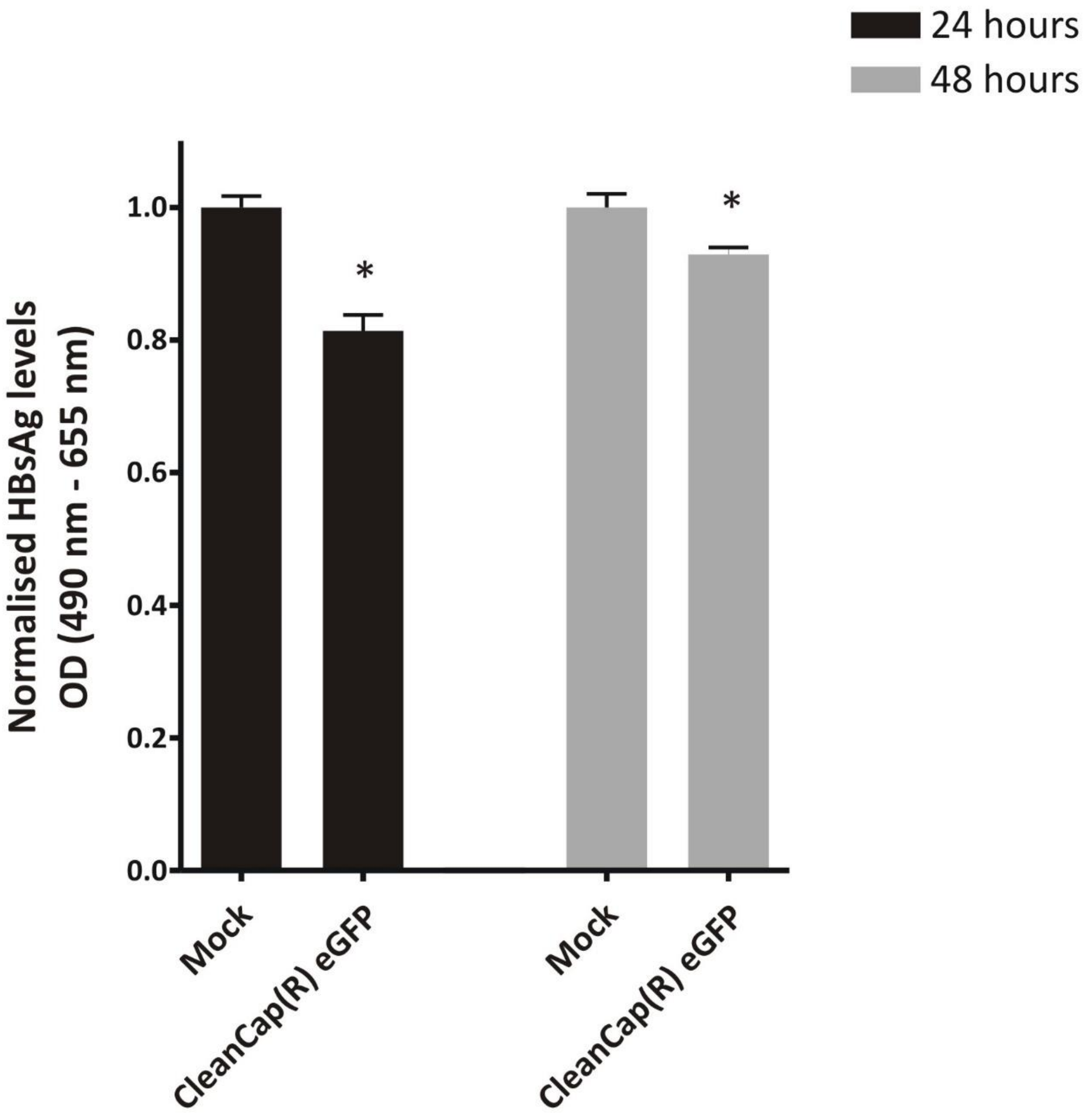

Supplementary Figure S2: Transfection of commercial mRNA reporter (HPLC purified).

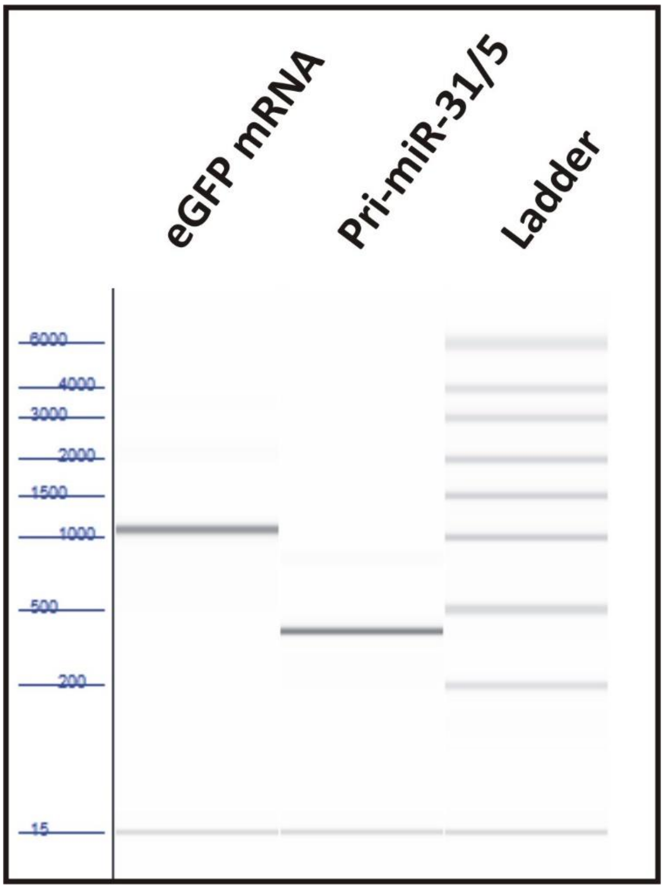

Supplementary Figure S3: Integrity of in vitro transcribed, capped RNA. Linearised eGFP and pri-miR-31/5 vectors were used as template for in vitro transcription. The RNAs were purified by cellulose chromatography and capped enzymatically using the Vaccinia Capping Enzyme System (NEB). RNAs were analysed by Fragment Analyzer (Agilent) (eGFP mRNA = 959 nt; pri-miR-31/5 = 387 nt).

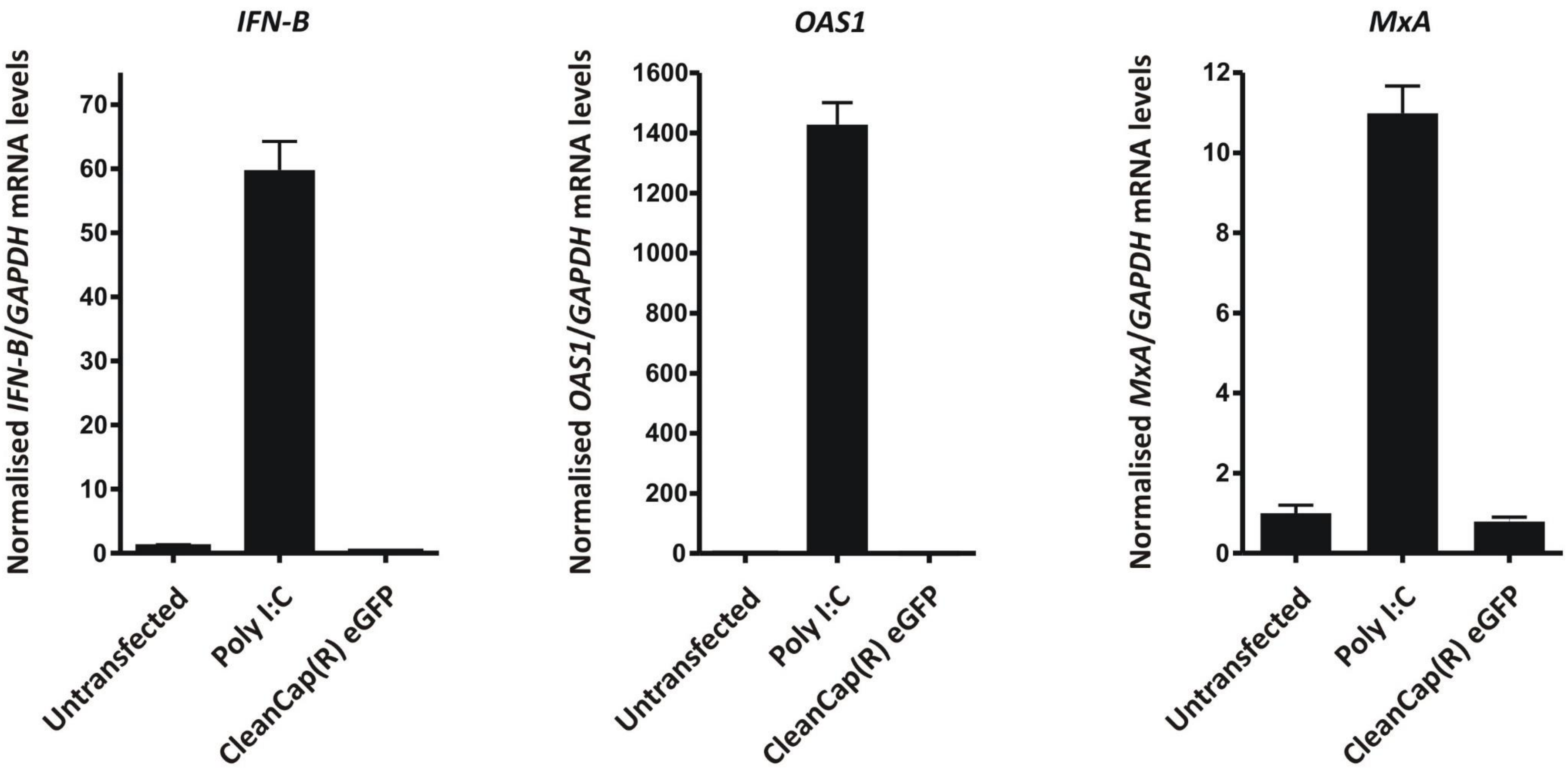

Supplementary Figure S4: RT-qPCR analysis of interferon gene induction. Induction of interferon response by commercial eGFP mRNA (HPLC purified).
